# Survival study of leukoplakia malignant transformation in a region of northern Spain

**DOI:** 10.4317/medoral.22326

**Published:** 2018-06-21

**Authors:** Pilar Gandara-Vila, Mario Pérez-Sayans, Jose M. Suárez-Peñaranda, Mercedes Gallas-Torreira, Jose Somoza-Martín, Maria-Dolores Reboiras-López, Andrés Blanco-Carrión, Abel García-García

**Affiliations:** 1 PhD, DDS. Oral Medicine, Oral Surgery and Implantology Unit. Faculty of Medicine and Dentistry, Santiago de Compostela, Spain; 2 PhD, DDS. Oral Medicine, Oral Surgery and Implantology Unit. Faculty of Medicine and Dentistry. Instituto de Investigación Sanitaria de Santiago (IDIS), Santiago de Compostela, Spain; 3 MD, PhD. Department of Pathology and Forensic Sciences. University Hospital and School of Medicine of Santiago de Compostela; 4 PhD, DDS. Oral Medicine, Oral Surgery and Implantology Unit. Faculty of Medicine and Dentistry. Santiago de Compostela, Spain; 5 MD, PhD. Oral Medicine, Oral Surgery and Implantology Unit. Faculty of Medicine and Dentistry. Instituto de Investigación Sanitaria de Santiago (IDIS), Santiago de Compostela, Spain

## Abstract

**Background:**

Oral leukoplakia is the most common potentially malignant disorder (PMD) of the oral cavity. The objectives of this study are to determine the clinicopathologic features in a group of patients with oral leukoplakia of Northern Spain (Galicia), determining the factors associated to clinical risk and analyzing the malignant transformation of these patients.

**Material and Methods:**

We included 85 patients. We recorded sex and age, habits like alcohol and tobacco, size, clinical appearance, site, number of lesions, and presence or absence of dysplasia. We assess the association between risk factors and transformation and developed a logistic regression analysis. Finally we used the Kaplan-Meier and log-rank test for the survival analysis.

**Results:**

7 patients (8.2%) had malignant transformation. The mean follow-up of the patients was 4.13 years versus 5.58 years of those who developed carcinoma. Only location and initial dysplasia have a statistically significant relationship with malignant transformation, but when applied the long rank test only the presence of dysplasia remains statistically significant(*P*<0,026). Oral Cancer Free Survival was 81.9% (0.150) at 11 years for the group without dysplasia.

**Conclusions:**

We found that the presence of dysplasia is the only risk factor that is statistically related to the development of a carcinoma.

** Key words:**Leukoplakia, oral cancer and oral precancer, follow-up, malignant transformation.

## Introduction

Oral leukoplakia is the most common potentially malignant lesion of the oral cavity, it is estimated that the overall prevalence is 1.72-2.60% (1). It is defined as “a white plate of questionable risk, having excluded (other) known diseases or disorders that do not increase the risk of cancer” (2).

Most leukoplakia lesions are benign lesions. A percentage of these acquire progressive dysplastic changes (3) and finally can result in the development of a carcinoma (4). The percentage of malignancy varies from one study to another, reaching a range from 0.13% (5) to 34% (6); if we consider only the form of proliferative verrucous leukoplakia can reach 70.3.% (7). Today we still don’t have the means at our disposal that allows us to know the prognosis of an individual lesion (8,9).

In literature there are certain risk factors associated with malignant transformation such as advanced age (3,10,11), female sex (12), idiopathic leukoplakia (non-smokers) (11,13), long evolution (14), size above 200mm2 (15,16) or length of major axis of more than 4 mms (17), non homogeneous clinical form (11,18,19), the subsite of tongue and floor of the mouth (20) and the presence of dysplasia (10,21-23). But the results vary when we compare different studies.

The objectives of this study are to determine the clinical and pathological features of leukoplakia of the oral cavity in our area in a group of 85 patients from Northern Spain (Galicia), and identifying the factors associated to malignant transformation and doing a study of survival of patients with this disease.

## Material and Methods

-Patients

Patients clinically diagnosed with oral leukoplakia were selected from the database of patients who attended the Oral Medicine Unit Clinic of the School of Dentistry at the University of Santiago de Compostela between January 1995 and June 2010.

This study complied with the Helsinki Declaration. The study received the approval of the ethics committee of the Galician Department of Health .

-Inclusion and exclusion criteria

Patients with clinical leukoplakia lesions (as defined by the WHO (2) in 2005:” white plaques of questionable risk having excluded (other) known diseases or disorders that carry no increased risk for cancer”) were included, such cases were confirmed by incisional or excisional biopsy (diagnostic certainty 3 and 4, using Van der Waal’s classification), of which we obtained access to the clinical history, clinical images of the lesion and the anatomic-pathological material.

Exclusion criteria were: 1) Lack of initial histological diagnosis; 2) follow-up period of less than one year; 3) lesion with clinical appearance or histological findings white, or predominantly white, benign disease such as leukoedema, linea alba, potentially malignant lesions such as lichen planus, discoid lupus erythematosus, etc. 4) lack of initial clinical image of the lesion; 5) lack of access to the histological material allowing for confirmation of the initial diagnosis; 6) presence of a concomitant carcinoma or diagnosed within 6 months after the initial diagnosis.

Of the 90 patients who met the inclusion requirements established for the study, we excluded 5 who showed a carcinoma in the first biopsy or who were diagnosed with one in less than 6 months after their first visit.

-Clinical data

Demographic data (sex and age) and clinical data relevant to leukoplakia (location (according to the ICD-10 recommendations (2,24) were recorded, as well as the clinical form following the WHO’s classification (2,24). In order to determine the size of the lesion following Van der Waal (25,26), we recorded the size of the lesion based on the length of the major axis, thus differentiating three groups (0-2 cms, 2-4cms and >4 cms), and finally, single or multiple location (affecting more than one subsite). All these data were collated from the clinical records, in some cases the form and size were determined using clinical photograps from the lesions.

The information on toxic habits, such as smoking (27), we divided them into two groups, smokers and non-smokers, and included in the former current smokers and those who had been smokers in the past 10 years; and in the latter, those who have never smoked or have quit over 10 years prior. As for alcohol, we decided to classify patients as a drinkers and non-drinkers, since in most medical records the amounts are not registered.

Follow-up was performed every 6 or 3 months, depending on the characteristics of the lesion, to assess progress.

-Histopathologic examination 

All patients in the study underwent biopsies at the time of initial diagnosis and, in some cases, during the follow up, if changes that were likely to become malignant were observed.

The biopsied tissue was fixed in 10% formaldehyde, subsequently embedded in paraffin and processed for routine histological study using the hematoxylin-eosin technique.

Once the tissues were collected, all cases were assessed again by the same oral pathologist and further confirmed by an independent observer.

Gradation of dysplasia was evaluated according to the criteria of the WHO (28) and classified as mild, moderate and severe. For the survival study, we transformed this variable into a binary: absence / presence of dysplasia.

-Statistical study

All data collected was entered into a database using Microsoft Office Excel 2007 for Windows. Subsequently, the data was tabulated and analyzed statistically using SPSS software for Windows, version 20.0 (SPSS Inc., Chicago, IL, USA).

We carried out a descriptive analysis of clinical and pathological factors. In order to compare means, we used the t student test or the ANOVA single-factor test.

To assess the association between risk factors and transformation, we transformed the variables into binary options and used the Pearson chi-square test or Fisher’s exact test, when the frequency for a given variable was less than 5. We also developed a logistic regression analysis to assess the effect of risk.

For the survival analysis, we used the Kaplan-Meier and log-rank tests to assess if the generated survival curves were significantly different. The study time was considered the time from the completion of the first biopsy to the development of cancer (non-censured observation) or until the end of the patient follow-up (censored observation).

All *p*-values (P) are bilateral, and *p*-values of 0.05 or less are considered as significant.

## Results

-Patient characteristics 

The group of 85 patients consisted of 45 men and 40 women, with an average age of 58.68 years (SD = 12.88, and range from 22 to 85).

The most frequently affected site was the tongue (37.6%) followed by gum and buccal mucosa, both in 22.4% of patients. 57.6% of leukoplakia were non homogeneous and, within these, nodular leukoplakia (29.4%) was the most common and 55.3% had more than one affected location. For the initial diagnosis, 62.4% of the biopsies were incisional, while 37.6% were excisional. 44.7% of patients were smokers, the mean daily consumption was of 24.08 units (SD ± 16.63), with minimum and maximum values of 2 and 100, respectively, the case of the smoker accounting for two units he smoked cigars. 73.7% were heavy smokers (smoking more than 20 cigarettes). Smoking was more frequent in the group of men (73.7%). As for alcohol consumption, this information was not included in the medical history of 7 patients; of the drinkers (35.9%), men accounted for 67.9% contrasting with 32.1% women ( Table 1).

**Table 1:**
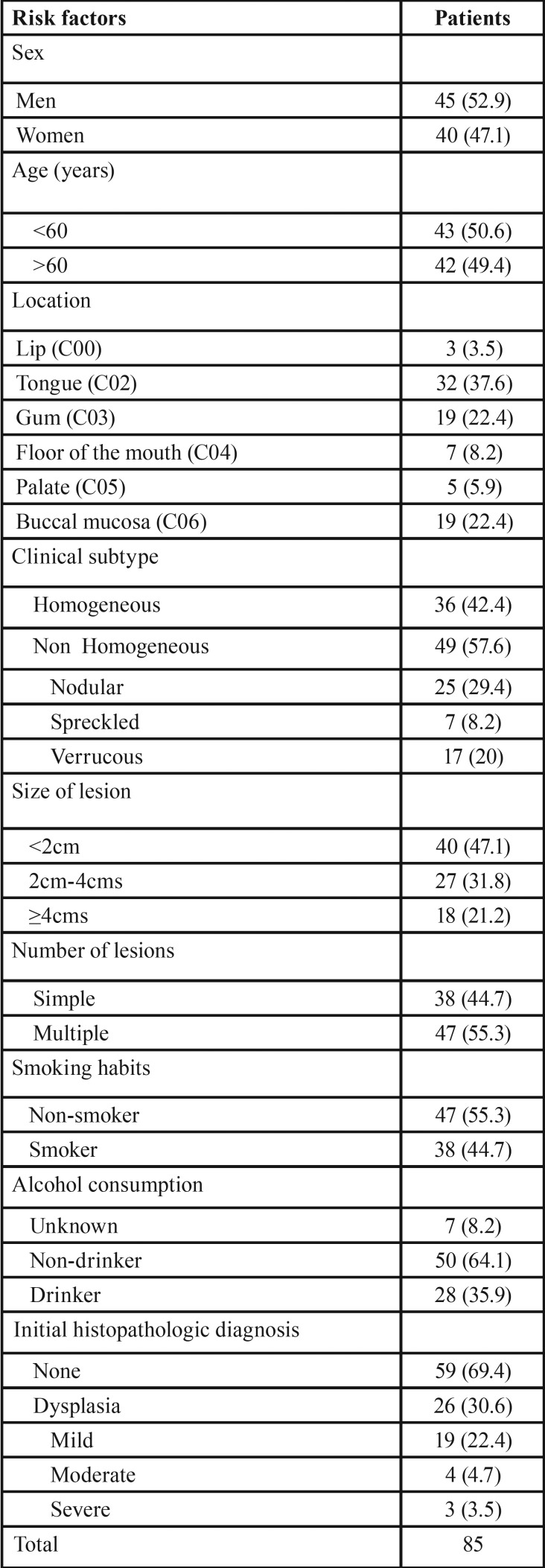
Characteristics of the studied population.

-Clinical characteristics and relationship with malignant transformation

Table 2 presents the results from comparing the clinical characteristics between patients who do not suffer transformation and those who did. The men/women affectation ratio was 1.1 / 1 for the group in general, when assessing the number of cases that have become malignant, the ratio is reversed to 1: 1.3, with women suffering from higher levels of malignant transformation.

The mean age at end of follow-up was 64.14 (95% CI (53.76 to 74.53)) for the group that develops carcinoma and the average age in the group that does not develop the event at the end of follow-up was 62.69 (95% CI (59.68 to 65.69)). When applying the ANOVA mean comparison test, we note that there are no differences between the two groups.

 Table 2 shows that only two of the risk factors: location and initial histological diagnosis have a statistically significant relationship with malignant transformation. The tongue and floor of mouth are the preferred locations of malignancy as well as patients with dysplasia at diagnosis. In patients without dysplasia at the beginning of the treatment, we must emphasize that one developed cancer 11 months after the first visit, He was a 38 year old male, who smoked over 20 cigarettes per day, which contrasts with the other case, a non-smoking 58.8 year old woman who developed a carcinoma 11 years later.

**Table 2:**
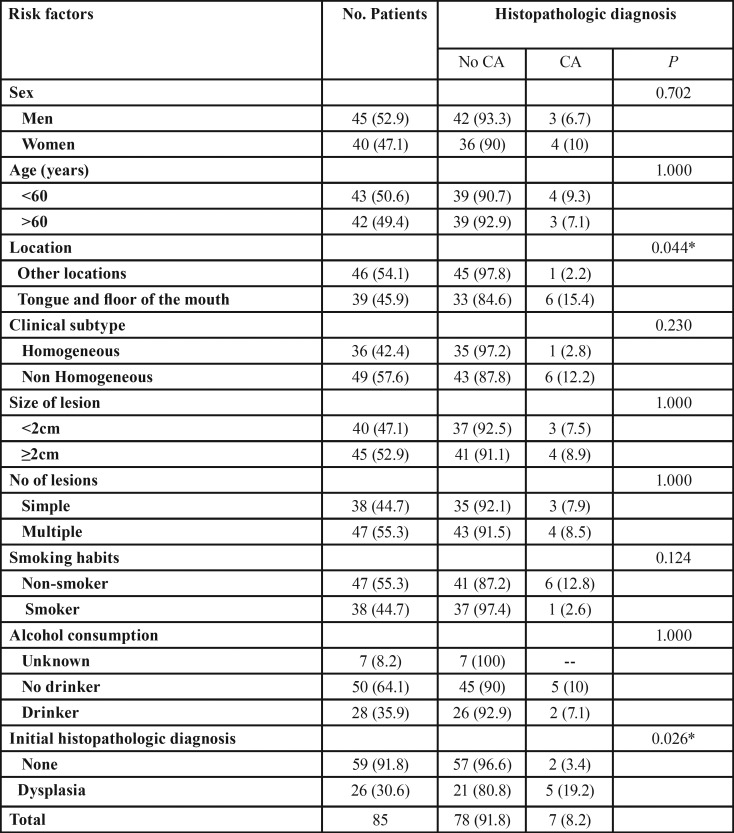
Distribution (%) of risk factors and histopathologic diagnosis of the 85 patients and p-values of risk comparison.

Of the 7 patients who suffered malignant transformation, carcinoma develops in areas of previous lesions except in a case in which leukoplakia appears in the lateral surface of the tongue and the carcinoma develops in the ipsi-lateral alveolar ridge and there was no previous lesion. Two other cases where the initial biopsy was taken at one location and a carcinoma is developed in a different location but is affected by leukoplakia.

-Histopathologic studies of samples

Of the leukoplakia cases showing dysplasia at the initial visit: 19 were mild (21.1%), 4 moderate (4.4%) and 3 severe (3.3%).

-Survival analysis

In our study, 7 (8.2%) of the 85 patients had malignant transformation. The mean follow-up of the 85 patients was 4.13 years (95% CI: (3.36 to 4.89)) versus 5.58 years (95% CI: (1.15 to 10.02)) of those who developed carcinoma.

**Figure 1:**
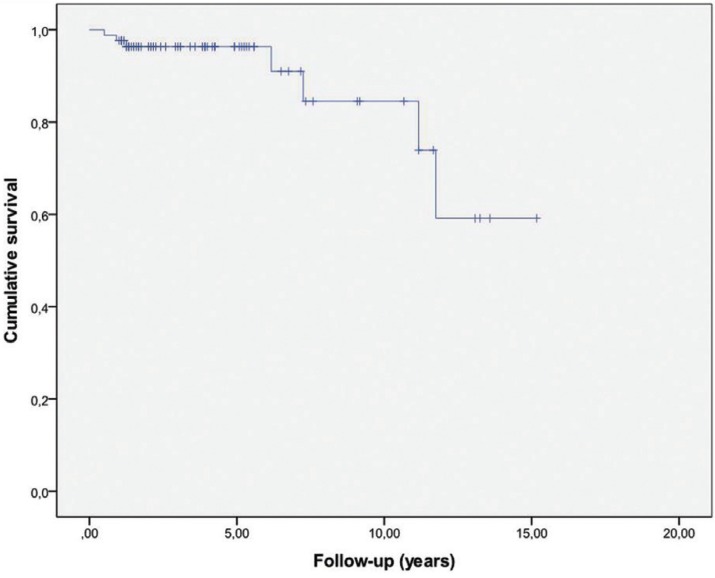
Kaplan Meier curve analysis of malignant transformation.

As we can observe in Figure 1, the Kaplan Meier survival curve for all lesions of the general group without distinction by risk factors. We can see how most of the transformations take place from 5 years of follow-up onwards.

In  Table 3 we see the proportion of malignant transformation free survival (OCFS) during the follow-up at 1, 5, 7, 8 and 11 years. Thus, we see that at 5 years, 96.3% of the sample was free of transformation. The rate of malignancies at 5 years was 3.7%, as the follow-up period increases, so does the number of transformations.

**Table 3:**
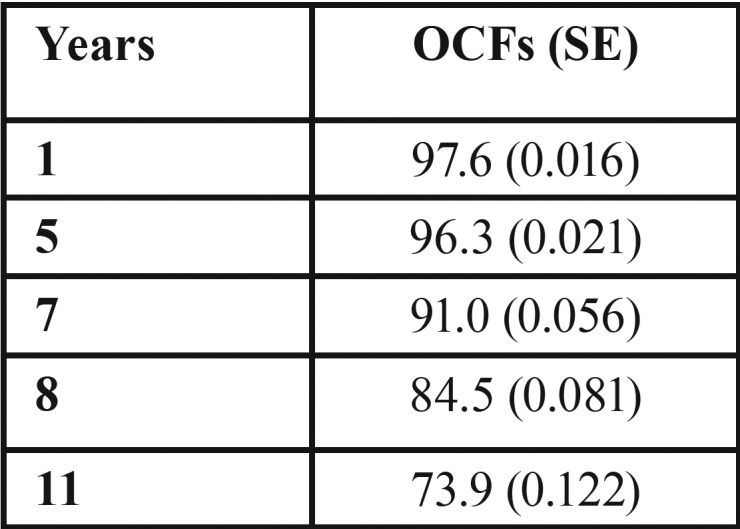
Proportion of oral cancer free survival (OCFS), in which SE is standard error, throughout the check-ups at 1, 5, 7, 8, and 11 years.

We can also see how OCFS takes different values when calculated according to the degree of dysplasia and location ( Table 4). In patients without dysplasia we have estimated an OCFS at five years of 98.3% (0.017) compared to 92.1 (0.053) of the group having dysplasia. For other factors, considering them as independent variables, we conducted a study of binary logistic regression to measure risk, considering malignant transformation as a dependent variable ( Table 5).

**Table 4:**
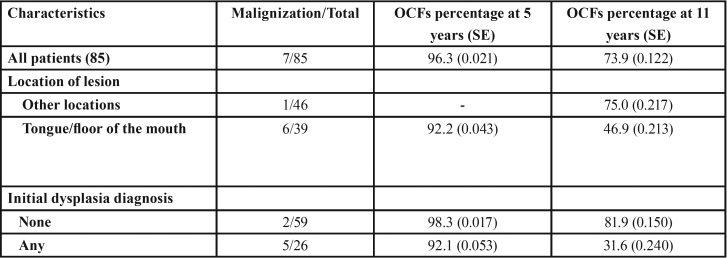
Oral Cancer Free Survival (OCFs) by degree of dysplasia and location.

**Table 5:**
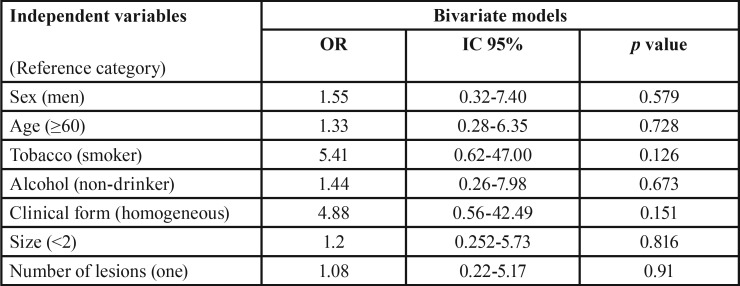
Analysis of logistic regression of the risk of developing carcinoma for independent variables.

**Figure 2:**
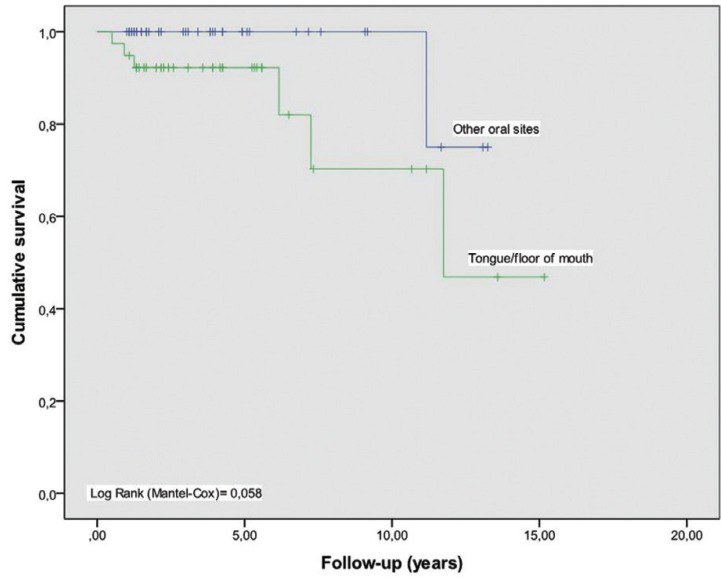
Kaplan-Meier curve for location and period free from malignant transformation.

**Figure 3:**
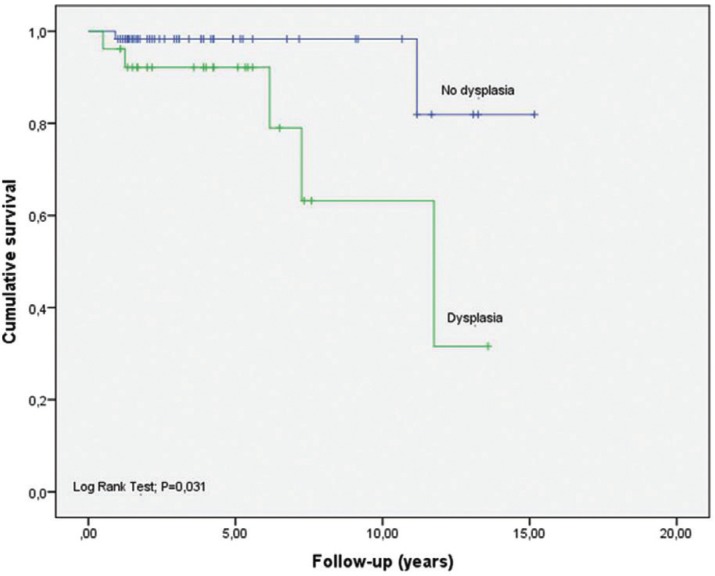
Kaplan-Meier curve for dysplasia and period free from malignant transformation.

In Figures 2 and 3, survival curves regarding these risk factors: localization and dysplasia. By comparing the curves with the log rank test, the significance in terms of location (*P* = 0.058) disappears, in which only the presence of dysplasia remains statistically significant (*P* = 0.026).

Therefore, in our samples, we found that the presence of dysplasia is the only risk factor that is statistically related to the development of a carcinoma.

## Discussion

Precancerous lesions, leukoplakia in particular, is a difficult field of study due to changes in the nomenclature and definition since they were first described until now. Also, there are no established criteria for epidemiological study, so we find recommendations (25,29-31).

Many authors studied oral dysplasia regardless of the clinical lesion from which the samples were taken from, treating all potentially malignant disorders equally. Few articles carried out the study of oral leukoplakia lesions exclusively and consider only dysplasia from it (4). If we know that the lesion having higher chances of malignization is leukoplakia, we should not treat all dysplasias equally, because in this way we could draw erroneous conclusions regarding the behavior of a particular clinical lesion. Even within leukoplakia we can see different behaviors depending on the consumption habits related to their etiology, as is the case of consumption of betel nut, very rare in our society, which is a carcinogenic factor in itself, or in the case of smoking, it would be necessary to establish a criteria first with respect to a classification by smoking habits.

For this reason, we have solely analyzed the lesions that meet the clinical criteria of leukoplakia with anatomic-pathological confirmation.

The rate of malignant transformation, when evaluating all the leukoplakia, regardless of the degree of dysplasia appearing varies widely from 0.13% to 17.9% (5,13,18,23,32,33). In our study, 7 of the 85 patients suffered malignant transformation representing a transformation ratio of 8.2%. Other authors found similar indices such as Amagasa (10) with a rate of 7.9%.

Regarding the mean follow-up in our group, it was 4.14, while it amounted to 5.58 years in those patients who suffered malignant transformation. With these results we can conclude that the longer the follow-up time the higher the risk of transformation into carcinoma, as has been shown by other studies (11,13). Authors such as Silverman found a conversion ratio of 6% in its first research and by increasing the follow-up period it increased to 17.5% (13). Lummerman (34) observed a transformation of dysplastic lesions in 33.6 months and Ho MW (16) observed a mean follow-up time of 48 months, from the first visit to the transformation.

Most studies described cases of appearance of carcinomas that were distant from the initially described leukoplakia (3,11,34). Some authors also describe remote malignizations far from the areas affected by leukoplakia in oral mucosa of normal appearance (6,34), all facts supporting the theory of field malignant transformation.

Like other authors (6,34), we found a case of a patient who developed cancer in a remote location of normal-appearing mucosa that was not affected by leukoplakia. Lee (3) observed that in 41% of patients the cancer was developed in areas far from the initial lesion, this can be explained by the theory of field cancerization (35), according to which any area of mucosa exposed to carcinogens can develop premalignant changes. Thomson (36) found dysplastic areas in remote areas of the initial lesion and with the appearance of normal oral mucosa in 15 patients. In contrast to our results, Brouns (17) describes that all carcinomas arose in the same location as the initial leukoplakia.

The malignant transformation free period or Oral Cancer Free survival (OCFS) at 5 years was 96.3 (0.021) and 73.9 (0.122) at 11 years. Very few studies calculating the rate of transformation in OCFS, which would be ideal to compare populations, thus we should consider the follow-up time of patients so that the rate of malignant transformation is time-person dependent. We only agreed with Liu (18) in the malignant transformation free period, which differentiates when initial lesions are low risk or high risk, and found a low risk OCFS of 90.6% (95% CI 0.86-0.96) and high risk 61.7% (95% CI 0.44-0.80). As in our study, when we consider the dysplasia-free group we found a greater OCFS than in patients with dysplasia.

The distribution by sex shows a male / female ratio of 1.1:1 similar to other studies (20,34,37). However, this index is inverted when we analyze the cases of malignization, 1: 1.3 (men/women), despite this we did not find statistically significant differences in malignization like other authors (17,38), but we did find a higher proportion of women who developed carcinoma (6,17,23).

No relationship between the location of the leukoplakia and a greater transformation was found, although the statistically significant association between localization in the tongue and malignization is described in the literature (23). We must keep in mind that most OSCC cases arise from the floor of the mouth, the soft palate and the ventral / lateral tongue surface (39). Epithelia lining the high cancerization risk areas are associated with dysplasia more frequently (40). The trend of dysplasia has been associated to high risk areas to include genetic alterations (loss of heterozygosity LOH at 3p and/or 9p) with a high risk of progression (41) .

## Conclusions

The clinicopathological characteristics of the studied leukoplakia in our area were consistent with those found by other authors from countries of Western Europe. We found that non homogeneous lesions pose a risk of developing a carcinoma almost 5 times higher compared to homogeneous lesions Although initially it seemed that the two factors related to the malignant transformation of leukoplakia were the subsite and the presence of dysplasia, ultimately the only factor clearly associated with malignant transformation is the presence of dysplasia of any kind either mild, moderate or severe. The survival analysis results underline the importance of comprehensive monitoring of these patients and the need to carefully observe any changes that occur in any area of the oral cavity of the patients diagnosed with leukoplakia.
